# Investigating Users' and Other Stakeholders' Needs in the Development of a Personalized Integrated Care Platform (PROCare4Life) for Older People with Dementia or Parkinson Disease: Protocol for a Mixed Methods Study

**DOI:** 10.2196/22463

**Published:** 2021-01-12

**Authors:** Mona Ahmed, Mayca Marín, Raquel Bouça-Machado, Daniella How, Elda Judica, Peppino Tropea, Ellen Bentlage, Michael Brach

**Affiliations:** 1 Institute of Sport and Exercise Sciences Münster University Münster Germany; 2 Asociación Parkinson Madrid Madrid Spain; 3 Campus Neurológico Sénior Torres Vedras Portugal; 4 Department of Neurorehabilitation Sciences Casa di Cura del Policlinico Milan Italy

**Keywords:** dementia, older adults, neurodegenerative diseases, integrated care, health care technologies, user-centered design

## Abstract

**Background:**

Dementias—including Alzheimer disease—and Parkinson disease profoundly impact the quality of life of older population members and their families. PROCare4Life (Personalized Integrated Care Promoting Quality of Life for Older Adults) is a European project that recognizes the benefit of technology-based integrated care models in improving the care coordination and the quality of life of these target groups. This project proposes an integrated, scalable, and interactive care platform targeting older people suffering from neurodegenerative diseases, their caregivers, and socio-health professionals. PROCare4Life adopts a user-centered design approach from the early stage and throughout platform development and implementation, during which the platform is designed and adapted to the needs and requirements of all the involved users.

**Objective:**

This paper presents the study protocol for investigating users’ needs and requirements regarding the design of the proposed PROCare4Life platform.

**Methods:**

A mixed qualitative and quantitative study design is utilized, including online surveys, interviews, and workshops. The study aimed to recruit approximately 200 participants, including patients diagnosed with dementia or Parkinson disease, caregivers, socio-health professionals, and other stakeholders, from five different European countries: Germany, Italy, Portugal, Romania, and Spain.

**Results:**

The study took place between April and September 2020. Recruitment is now closed, and all the data have been collected and analyzed in order to be used in shaping the large-scale pilot phase of the PROCare4Life project. Results of the study are expected to be published in spring 2021.

**Conclusions:**

This paper charts the protocol for a user-centered design approach at the early stage of the PROCare4Life project in order to shape and influence an integrated health platform suitable for its intended target group and purpose.

**International Registered Report Identifier (IRRID):**

DERR1-10.2196/22463

## Introduction

### Background

Among the common chronic diseases, dementias—including Alzheimer disease (AD)—and Parkinson disease (PD) are the most disabling, profoundly impacting the quality of life of older population members and their families [1]. As a result, European health care systems are being challenged by the increasing demand and need for long-term care and services as well as increased health costs [2,3]; therefore, finding and implementing alternative health care solutions to face these challenges are needed [4].

Integrated care is defined as “services that are managed and delivered so that people receive a continuum of health promotion, disease prevention, diagnosis, treatment, disease management, rehabilitation and palliative care services, coordinated across the different levels and sites of care within and beyond the health sector, and according to their needs throughout the life course” [5]. Integrated care is able to help improve care coordination and decrease the costs of care for populations with complex needs [2]. This shift in the type of care for older people does not intend to neglect good disease management but rather to optimize older people’s physical and mental capacities [6]. On the other hand, assistive technologies can help older people with chronic conditions to live independently for a longer period [7], reduce caregiver burden, and improve the work satisfaction of professional carers [7-11]. Recent studies reveal that patients identify technology as something useful that can be used for leisure and to increase their freedom and independence. Caregivers find that technology incorporated into their lives, and into the lives of the people they care for, provides them with increased peace of mind and can ease the interactions with other stakeholders [12]. Furthermore, health professionals consider that technologies reduce their workload and allow them to devote more attention to patients who require it [13].

### PROCare4Life Project

Personalized Integrated Care Promoting Quality of Life for Older People (PROCare4Life) is a European Commission Horizon 2020 project (grant agreement No. 875221) that recognizes that, today, an integrated care process should be adopted for harmonizing, from a holistic perspective, health models with social services. Therefore, the project intends to develop and test a technology-based, integrated, scalable, and interactive care platform addressed to patients suffering from neurodegenerative diseases, such as PD or dementias; their caregivers; and socio-health professionals. The aims of this 36-month project are as follows: (1) to improve the quality of life of patients, (2) to enable active living and better disease management, (3) to support professionals in the decision-making process, (4) to facilitate efficient communication between all stakeholders, and (5) to ensure reliable and protected access to data within Europe. These aims will be achieved through large-scale assessments across Europe (ie, more than 1500 end users from five countries), with a final scope to validate the reliability of the overall system in a real-life context.

The technology of the PROCare4Life platform works through different components as follows:

1. Sensing component, which is responsible for patients’ data acquisition through a set of employed sensors (eg, audio, depth, and presence sensors and wearable devices); these sensory data are first preprocessed and filtered before feeding into the next component.
2. Low-level analysis component, where the data received from the sensing component are further processed and analyzed in order to recognize individual disease-related symptoms, human behavior, and cognitive abilities.
3. High-level analysis component, where the input from the low-level analysis is fused with other personal information (ie, social and medical) in order to build a profile for each patient. This profile will be used to provide personalized recommendations regarding promotion of social networking, nutrition, leisure, and best daily activities for increasing well-being and improving physical and mental conditions. All the data developed within this component will be contained within a secure cloud environment.
4. The interaction component, where, through different services and interfaces, the generated information will be shown to the users via various digital devices (eg, mobile phone, tablet, and PC). This information can serve as notifications in the form of alerts or reminders or as a communication tool, providing a safe information channel for the social and health professional to access the patients’ profiles, read and provide reports, or receive updates about the patients’ conditions or, finally, as gamification, allowing the users to engage in motivating cognitive and functional games.

**Figure 1:**
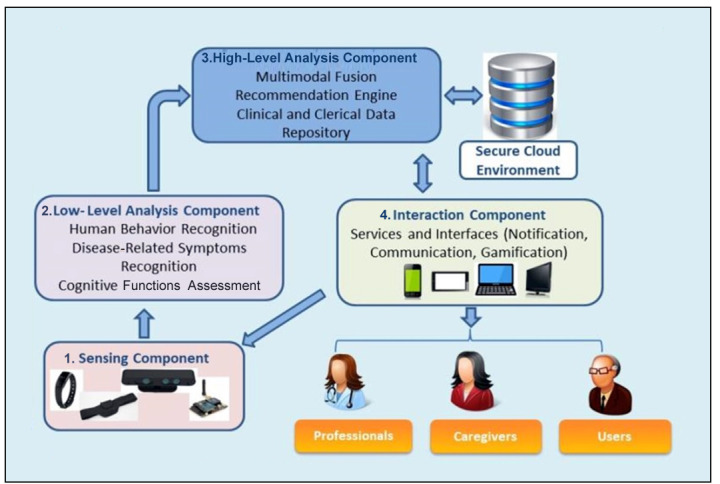
Technology of the PROCare4Life platform (source: PROCare4Life grant agreement, funded by The European Commission). PROCare4Life: Personalized Integrated Care Promoting Quality of Life for Older People.

PROCare4Life takes into consideration that when it comes to the inclusion of technology in health care systems, patients tend to have different priorities compared to caregivers and professionals [14]. Hence, the project adopts a user-centered design (UCD) approach, where all the end users are included rather than only the patients (see Figure 1).

### User-Centered Design

UCD is an iterative design process that advocates for actively engaging the users and incorporating their feedback to ensure that tools are developed with a full understanding of their needs and requirements [15,16]. The three key principles that underlie the UCD approach are as follows: (1) the design has to be based on the specific understanding of the users’ needs and requirements [17], (2) the design is refined and reshaped based on the user feedback throughout the whole development, and (3) the design process includes a multidisciplinary team, skills, and perceptions [18]. A number of studies have reported benefits from using UCD in the development of technology-assisted health measures. Bilodeau et al [19] reported that the UCD process facilitated optimization of comprehensiveness and relevance of content among a relatively small sample of dementia patients and their caregivers in shared decision making. Implementing a UCD process iteratively contributed to the development of an app for cancer screening, reflecting the needs and concerns of patients [20]. Also, the UCD process was recently implemented with success to develop a prototype for a digital cognitive aid in the anesthesiology emergency field, demonstrating high usability and user satisfaction [21].

Regardless of these promising results, health technology developers have been criticized for neglecting to incorporate UCD in the development process or for their failure in reporting the integration of a UCD approach throughout the development of their interventions. Possible reasons for this could be the limited resources and time to support the research and usability testing [22].

### Research on User Needs and Requirements

Integration of care services for older adults was proposed by the World Health Organization [23]. The Integrated Care for Older People (ICOPE) approach emphasizes the need for integrated health care models to be organized, coordinated, and delivered around older adults’ needs, preferences, and goals in the context of their daily lives as a family or community member [6,24]. In fact, in developing a useful and successful technological intervention that meets the needs and requirements of the end users, it is crucial to involve them at an early stage of the design process [25,26].

A review by Vermeer et al [27] indicated that research on the needs of older people living with dementia and their caregivers remained mainly qualitative, with more focus on the needs of caregivers than patients and a clear difference in perspectives. For instance, results from focus groups revealed that dementia patients had some concerns over the use of trackers, while their caregivers had a great interest in their use [28].

A previous information and communication technology (ICT)–based European project named ICT services for Life Improvement for the Elderly (ICT4Life) was funded by Horizon 2020 (grant agreement No. 690090). This project aimed to provide an integrated ICT platform that was able to facilitate integration of health care services and provide better communication between AD and PD patients, their caregivers, and socio-health professionals. ICT4Life had an end-user approach, where the needs and the expectations of the target population were considered throughout the development of the platform, starting with research on users’ requirements. While the use of the ICT4Life platform was accompanied with better and more personalized care for elderly patients with cognitive impairments [29], the research on users’ requirements involved limited study sites and a very low number of participants [30].

Adopting a UCD approach, PROCare4Life intends to build upon what has already been learned and to benefit from the knowledge gained from previous projects. Therefore, the project devoted its first phase to study user requirements and to investigate the key personal, social, health, and psychological factors that influence the daily lives of potential end users. The aim of this project phase is to define users’ needs and expectations regarding the use of technology within the integrated health care system. Collected data shall be used in the setup of the iterative cycles in the pilot phases, allowing a user-centered cocreation of a platform that supports patients, caregivers, and socio-health professionals in their functions.

Research on users’ requirements aims to involve approximately 200 subjects and is planned to take place in five different European countries: Germany, Italy, Portugal, Romania, and Spain. Surveys and interviews shall yield different perspectives. Preliminary results shall flow into workshops utilizing the interaction of potential users with the platform to evaluate and structure its aspects. The overall objectives are as follows:

1. Collection of detailed information on the opinions, thoughts, experiences, and feelings of users (eg, patients, caregivers, and other stakeholders, including socio–health care professionals) regarding their needs and difficulties during the health care process, to identify how a technology system such as PROCare4life can fit their needs and meet their demands.
2. Identification of those aspects that the PROCare4Life platform should consider in order to achieve success in its acceptance, development, and marketing (eg, strengths and weaknesses, factors that influence the digital health care market, and communication channels through which to adequately diffuse the product).

This paper aims to present the study protocol for investigating users’ needs and requirements regarding the design of the proposed PROCare4Life platform.

## Methods

### Study Design

**Figure 2:**
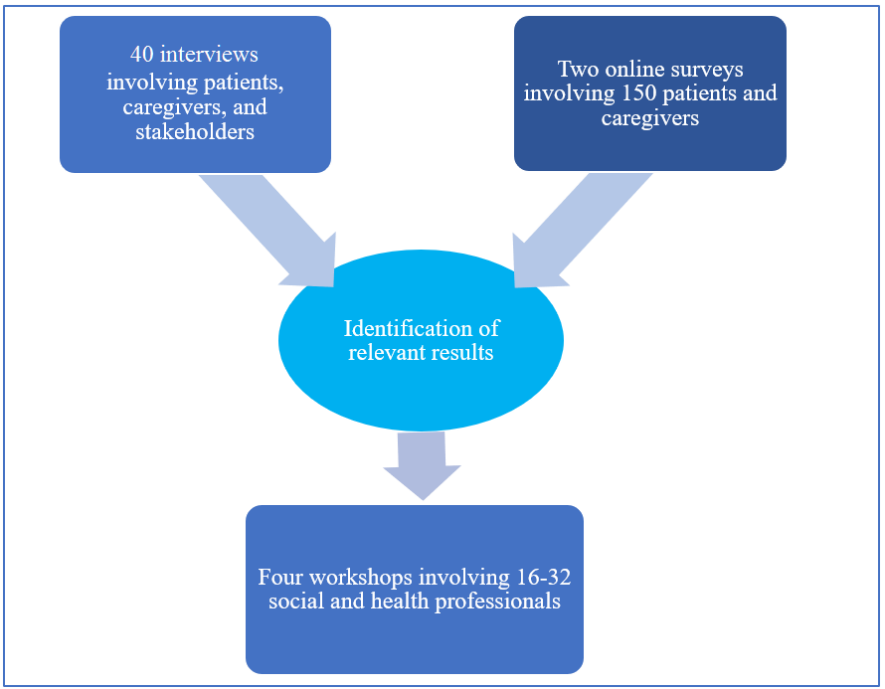
Mixed methods study design. The study started by conducting surveys and interviews; relevant results were identified and discussed in the workshops.

The study applied a mixed qualitative and quantitative research design and included different modalities of interaction, such as interviews, workshops, and online surveys. The study started by conducting online surveys and interviews; their relevant results were then discussed and analyzed by a multidisciplinary team in order to be used in reshaping the workshops (see Figure 2), which corresponds to the key principles of the UCD approach.

While quantitative research methods are used to provide general facts about a certain topic, qualitative research methods are more important for in-depth knowledge about the experiences and ideas of end users regarding health care issues [31]; qualitative methods have also been used as a way to engage patients and stakeholders in health research [32]. Using a mixed methods design allows the study to yield rich and comprehensive data that can better reflect the participants’ points of view [33] and thereby ensures the end users’ involvement in the project, which is a key principle of the UCD approach.

### Study Setting and Eligibility Criteria

The study started in April 2020 and ran until September 2020. It took place in five European countries: Germany, Italy, Portugal, Romania, and Spain. Patients were included if they were 65 years of age or older, clinically diagnosed with PD or dementia, and willing to participate in the study. Online survey participants should have a smartphone and/or computer with web access. Patients with significant cognitive impairment, intellectual disability, or other serious psychiatric conditions that affect their ability to use mobile phones or computers were excluded.

Caregivers were considered eligible if they were 18 years of age or older, caring for patients diagnosed with PD or dementia, formal (ie, paid) or informal (ie, not paid) caregivers, aware of the patients’ daily needs, and willing to participate. Online survey participants should have access to a mobile phone and/or a computer with web access.

Socio-health professionals were included if they were qualified and worked in medical or social care for PD or dementia patients (eg, physiotherapists, social workers, and occupational therapists), had a mobile phone or computer with web access, and were willing to participate.

Other stakeholders were included if they were in a responsible position within one of the following four categories:

1. Market actors: defined as those who work as health care providers either in public or private sectors.
2. Academic persons: people who work in research areas related to integrated care systems or assisted technologies for older adults.
3. Media actors: people who work for a relevant media outlet (eg, press, journalists, and bloggers).
4. Decision makers: policy makers and relevant health authorities.

### Recruitment

Each participant organization was assigned to recruit a number of participants for the different aspects of the study; this specific number was discussed and agreed on during the first project meeting, based on the effort expected from each organization related to their skills, experience, number of researchers involved, and access to users. The distribution of numbers was intended to ensure an equal distribution of all the countries involved in the project (see Table 1). Different recruitment strategies and channels were used as follows: (1) databases of the participating organizations, (2) national patient associations, (3) social networks (eg, Twitter, Facebook, and LinkedIn), (4) communication channels (eg, partner magazines), (5) face-to-face interactions by the nature of the organizations (ie, some hospitals, despite COVID-19, have allowed interaction with patients and caregivers), and (6) PROCare4Life’s website [34].

At every organization involved, the purpose of the PROCare4Life project was explained and the subjects who showed interest received information about the project either by phone or email. Subjects who met the inclusion criteria were asked to participate and were informed about the time frame of the study.

Due to COVID-19 security alerts in most of the European countries, all face-to-face interactions, such as interviews or group interactions (ie, workshops), were undertaken remotely via telephone or in online conference rooms. Virtual interaction ensured protection to all the stakeholders, while still engaging them in the UCD research, and allowed the project to go ahead with the large participant numbers.

**Table 1:** Distribution of participants per organization for each mode of data collection.

Name of organization	Country	Number of participants, n (%)^a^				
		Patient interviews (n=5)	Caregiver interviews (n=5)	Stakeholder interviews (n=30)	Workshops for health and social professionals (n=4^b^)	Online surveys (n=150)
Asociación Parkinson Madrid	Spain	2 (40)	1 (20)	2 (7)	1 (25)	25 (17)
Kinetikos	Portugal	N/A^c^	N/A	4 (13)	N/A	N/A
Campus Neurológico Sénior	Portugal	1 (20)	1 (20)	6 (20)	1 (25)	25 (17)
Casa di Cura del Policlinico	Italy	1 (20)	1 (20)	5 (17)	1 (25)	25 (17)
Wohlfahrtswerk für Baden-Württemberg	Germany	1 (20)	1 (20)	5 (17)	1 (25)	25 (17)
Münster University	Germany	N/A	N/A	6 (20)	N/A	N/A
Universitatea de Medicina si Farmacie "Carol Davila" din Bucureşti	Romania	N/A	1 (20)	2 (7)	N/A	25 (17)
Spitalul Universitar de Urgenţă Bucureşti	Romania	N/A	N/A	N/A	N/A	25 (17)

^a^Percentages may not add up to 100% due to rounding.

^b^There were four workshops, with 4 to 8 participants each.

^c^N/A: not applicable; the respective participants from this organization did not participate in this mode of data collection.

### Data Collection

#### Online Surveys

Two anonymous open surveys were developed using the EUSurvey application; the two online surveys were available on the project’s official website [35], where a post was written explaining the purpose of the surveys. Both surveys were launched on May 27, 2020, and ran until July 31, 2020; they were also distributed using mailing lists to some local patients’ associations and network organizations, aiming to recruit 150 participants, including patients and caregivers. Each participant had to agree to provide their consent before starting the survey; after that, participants could choose either the patient or caregiver version. The patient version included the 36-item Short Form Health Survey (SF-36), which is a widely used questionnaire to evaluate health-related quality of life [36]. The caregiver version included the 7-item Zarit Burden Interview to measure caregiver burden [37,38]. Both versions included questions related to the key performance indicators to be achieved within the project (eg, feelings of safety; feelings of autonomy; perception of empowerment; improvement of social participation, mental condition, and/or physical condition; anxiety; depression; and fall reduction), as well as other questions about technology acceptance, willingness to pay for such technologies, and the desired features and functionalities to be included in the PROCare4Life platform. Participants were not obligated to answer all the questions; however, at the end of the survey they had to click *send*, otherwise their participation would not be considered. The surveys were available in different languages, including English, German, Spanish, Italian, Romanian, and Portuguese. If needed, more information about the project or how to fill in the survey was sent to the participants via email, text message, or video call.

#### Interviews

A total of 40 semistructured interviews were carried out—5 patients, 5 caregivers, and 30 stakeholders—in the period from June to July 2020. Some of the researchers involved in developing the questions have previously worked on another European ICT project named ICT4Life (see Introduction); as ICT4Life also included assessment of user needs and requirements, the researchers used their past experience in helping to develop the questions for this study. In addition to the sociodemographic questions, the main questions’ topics were about participants’ experiences with the process of care, technology usage and acceptance, and the desired characteristics and expected outcomes of the proposed platform. Patient and caregiver interviews included some closed-ended questions; in particular, those asking about care services, symptoms, and activities of daily living (ADLs). Using closed-ended questions has been recommended with older people suffering from dementia, as it helps to facilitate communication with this population [39].

Different versions of questions were developed; for instance, for patients, the main topics included disease-specific questions, patients’ problems regarding their symptoms and how these could affect their ADLs, their needs and experiences with the health care process, and their attitudes and opinions toward using technology in the health care process. Caregivers were asked about their working experience and how technology-assisted platforms such as PROCare4Life would influence their roles as caregivers.

For other stakeholders, depending on the category they belonged to, there were supplemental questions. For instance, specific questions for academic stakeholders were included regarding the current status of research on the topic of technology-assisted, integrated health care platforms. Decision makers were asked about the existing systems of care, their readiness for innovation, and barriers they may encounter in integrating this type of care solution.

**Figure 3:**
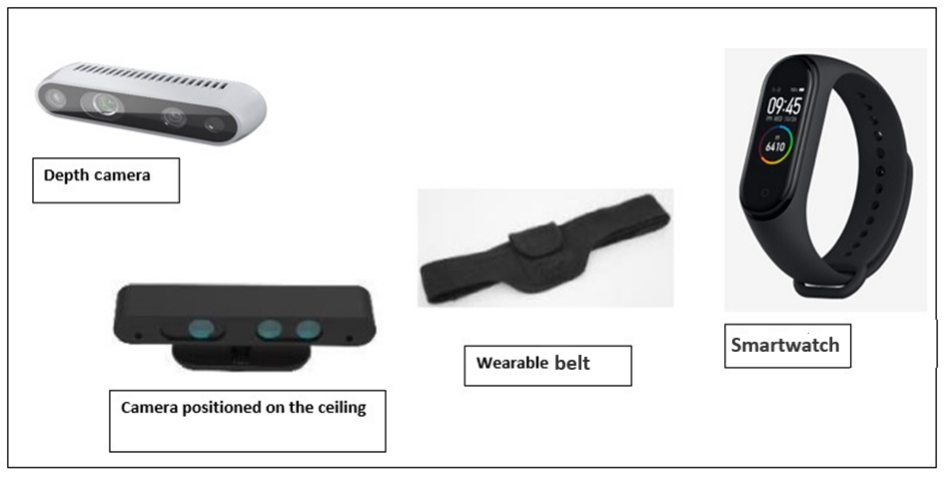
Example images of PROCare4Life tools and sensors viewed in interviews and workshops. PROCare4Life: Personalized Integrated Care Promoting Quality of Life for Older Adults.

Interviews included showing the participants some images of the devices and wearables (see Figure 3) to be used with the proposed platform along with a brief explanation, with questions aiming to identify their opinions regarding the platform features and functionalities.

#### Workshops

Four workshops took place between July and August 2020; each one included 4 to 8 socio-health professionals. The workshops have the advantage over other interview methods of both sharing questions in a group and allowing participants to test predefined parts of platform, such as mock-ups, drawings, and diagrams, allowing them to discuss these during the workshops to give feedback. In these workshops, relevant results obtained from the surveys and interviews were discussed and evaluated (see Figure 2).

### Data Analysis

#### Quantitative Analysis

Quantitative analysis was descriptive. The mean, median, standard deviation, and range of values were calculated. For categorical variables, absolute and relative frequencies were used to describe the data.

#### Qualitative Analysis

Researchers from the centers involved in the study transcribed the collected data from interviews and workshops and then translated them into English. Transcription templates have been provided using Microsoft Excel sheets and were made available for all involved partners. The analysis process was done by researchers from the Asociación Parkinson Madrid (APM) and Münster University following the thematic analysis approach [40,41], which involves six phases:

1. Familiarization with the data, which will involve reading the data and further organizing them with regard to the target groups and the question categories.
2. Acquiring identification codes.
3. Combining codes to generate different themes.
4. Reviewing themes in order to identify the recurrent themes; this phase also involves recreating, rearranging, or combining different themes together, aiming to make sense out of the data in relation to the research questions.
5. Defining and naming the themes, which will include checking the literature and relating the findings to other studies.
6. Finalizing the results with an explanation of the meaning and significance of the results along with reporting on the whole process of analysis.

The results were validated using a comparison of qualitative and quantitative data and through a discussion of the results with the end-user groups (eg, in the workshops). In addition, bias was avoided by analyzing qualitative data in two phases by researches from APM and Münster University.

### Ethical Consideration

#### Ethical Approval

The study protocol received approvals from the local ethical committees in Germany, Italy, Portugal, Romania, and Spain.

#### Data Handling and Management

The partners (ie, APM, Casa di Cura del Policlinico, Campus Neurológico Sénior, Wohlfahrtswerk für Baden-Württemberg, Spitalul Universitar de Urgenţă Bucureşti, and Universitatea de Medicina si Farmacie "Carol Davila" din Bucureşti). and the supporting partners (ie, Kinetikos and Münster University) established procedures and responsibilities for data protection management prior to the start of any processing of personal data, according to legal regulations and following good practice in research. In addition, they nominated responsible persons for data management from each study organization and each supporting organization.

#### Informed Consent

Once the study had been fully explained to the subject, written or digital informed consent was obtained prior to any study-related procedure, according to Good Clinical Practice and International Conference on Harmonization standards.

#### Security and Adverse Event Reporting

There were no direct physical risks for any of the participants in these interviews, workshops, and online surveys. There was a small risk of personal data theft; however, the PROCare4Life consortium received advice from professionals in the field of data management and protection and took all possible precautions to mitigate this risk, including encryption and secure storage.

#### Withdrawal of Participants

Participation in this study was entirely voluntary. Participants had the right to withdraw from the study at any time, without giving reasons or experiencing any disadvantage in terms of the quality of care they would receive if they did not participate. After the withdrawal, their data were not considered for statistical purposes. No replacements were considered.

## Results

Recruitment is now closed. All the data have been collected and analyzed in order to be used in shaping the large-scale pilot phase of the PROCare4Life project. Results of the study are expected to be published in spring 2021.

## Discussion

**Figure 4:**
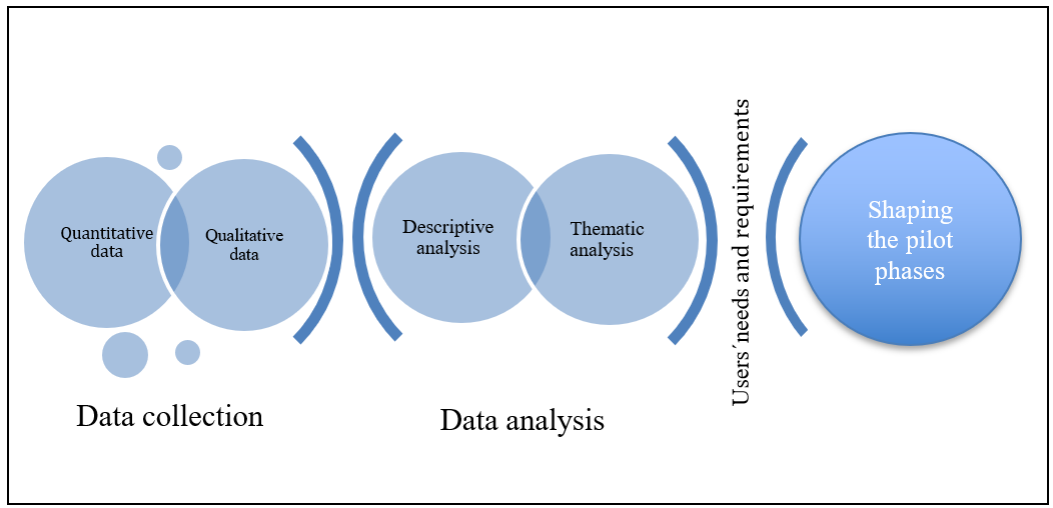
The process of investigating the users' needs and requirements. Qualitative and quantitative data were collected and analyzed using thematic and descriptive analysis methods. The identified results will be used to shape the pilot phases of the PROCare4Life project. PROCare4Life: Personalized Integrated Care Promoting Quality of Life for Older Adults.

Early involvement of end users assures the suitability of a product for its intended target group and purpose [42]. UCD allows users to shape and influence the product design, which can ultimately increase usability [22] and reliability [42]. PROCare4Life aims to apply the UCD approach from the early stage of the project until the last release of the proposed integrated care platform. The data gathered from the user requirements study will help the research team in designing the different modules and services of the platform (ie, deciding which technology should be developed and how it can be used effectively). The experience with the different methods used in the study will be valuable for preparing the iterative cocreation cycles of the pilot phases of the PROCare4Life platform (see Figure 4). Furthermore, we expect the gathered information to form the base of the development strategy for the final PROCare4life platform.

In conclusion, this manuscript reports on the protocol for a mixed methods study of users’ needs and requirements using the UCD approach for the European deployment of a personalized integrated care platform targeting older people with neurodegenerative disease, caregivers, and other stakeholders, including socio-health professionals.

## Abbreviations

AD: Alzheimer disease
ADLs: activities of daily living
APM: Asociación Parkinson Madrid
ICOPE: Integrated Care for Older People
ICT: information and communication technology
ICT4Life: ICT services for Life Improvement for the Elderly
PD: Parkinson disease
PROCare4Life: Personalized Integrated Care Promoting Quality of Life for Older Adults
SF-36: 36-item Short Form Health Survey
UCD: user-centered design
